# Predictive Signatures for Responses to Checkpoint Blockade in Small-Cell Lung Cancer in Second-Line Therapy Do Not Predict Responses in First-Line Patients

**DOI:** 10.3390/cancers16162795

**Published:** 2024-08-08

**Authors:** Jeffrey C. Thompson, Caitlin Tilsed, Christiana Davis, Aasha Gupta, Bihui Melidosian, Chifei Sun, Michael E. Kallen, Cynthia Timmers, Corey J. Langer, Steven M. Albelda

**Affiliations:** 1 Division of Pulmonary, Allergy and Critical Care Medicine, Thoracic Oncology Group, Department of Medicine, Perelman School of Medicine, University of Pennsylvania, 228 Stemmler Hall, 3450 Hamilton Walk, Philadelphia, PA 19104, USA; thompje84@gmail.com (J.C.T.); caitlin.tilsed@pennmedicine.upenn.edu (C.T.); aasha.gupta@pennmedicine.upenn.edu (A.G.); 2 Abramson Cancer Center, Perelman School of Medicine, University of Pennsylvania, Philadelphia, PA 19104, USA; christiana.davis@pennmedicine.upenn.edu (C.D.); corey.langer@pennmedicine.upenn.edu (C.J.L.); 3 Division of Hematology/Oncology, Department of Medicine, Perelman School of Medicine, University of Pennsylvania, Philadelphia, PA 19104, USA; 4 Incyte, Wilmington, DE 19803, USA; bmelidosian@incyte.com (B.M.); csun@incyte.com (C.S.); ctimmers@incyte.com (C.T.); 5 Department of Pathology, University of Maryland School of Medicine, Baltimore, MD 21201, USA; mkallen@incyte.com

**Keywords:** small-cell lung cancer, immunotherapy, biomarkers, immune checkpoint blockade, predictive biomarkers, antigen presentation

## Abstract

**Simple Summary:**

Although the high mutation burden in small-cell lung cancer (SCLC) suggests that it should be responsive to immune checkpoint blockade (ICB), relatively few patients have shown durable clinical benefit (DCB). To identify biomarkers of responses, biopsies from 35 patients treated with ICB (21 first-line and 14 second-line) were subjected to transcriptomic analysis and gene signatures were studied. Patients with DCB were compared to those with no clinical benefit (NCB). The response to ICB in the second-line, but not the first-line, setting was associated with gene signatures of inflammation, antigen presentation, interferon responses, and increased CD8 T cells. Our data suggest that responses to ICB in the second-line setting can be predicted by the baseline inflammatory state of the tumor; however, this strong association with inflammation was not observed in the first-line setting, likely because chemotherapy alters the immune milieu allowing a response to ICB.

**Abstract:**

Although immune checkpoint blockade (ICB) is currently approved for the treatment of extensive-stage small-cell lung cancer (SCLC) in combination with chemotherapy, relatively few patients have demonstrated durable clinical benefit (DCB) to these therapies. Biomarkers predicting responses are needed. Biopsies from 35 SCLC patients treated with ICB were subjected to transcriptomic analysis; gene signatures were assessed for associations with responses. Twenty-one patients were treated with ICB in the first-line setting in combination with platinum-based chemotherapy; fourteen patients were treated in the second-line setting with ICB alone. DCB after ICB in SCLC in the second-line setting (3 of 14 patients) was associated with statistically higher transcriptomic levels of genes associated with inflammation (*p* = 0.003), antigen presentation machinery (*p* = 0.03), interferon responses (*p* < 0.05), and increased CD8 T cells (*p* = 0.02). In contrast, these gene signatures were not significantly different in the first-line setting. Our data suggest that responses to ICB in SCLC in the second-line setting can be predicted by the baseline inflammatory state of the tumor; however, this strong association with inflammation was not seen in the first-line setting. We postulate that chemotherapy alters the immune milieu allowing a response to ICB. Other biomarkers will be needed to predict responses in first-line therapy patients.

## 1. Introduction

Small-cell lung cancer (SCLC), an aggressive neuroendocrine tumor characterized by rapid cellular proliferation and early metastases, represents approximately 15% of new lung cancer cases. The majority of patients have metastatic disease at presentation, with a median overall survival of only 10–13 months. The standard therapy for extensive-stage SCLC, up until recently, traditionally included platinum-based chemotherapy with etoposide. Although the disease is initially highly sensitive to treatment, relapses occur quickly, and most patients die of recurrent disease [1,2].

SCLC is strongly associated with smoking and carries a high mutational burden, suggesting that it should be responsive to immune checkpoint blockade (ICB). Initial studies in the second-line setting [3,4,5,6] suggested some efficacy with surrogate endpoints; however, longer follow-ups were less encouraging, and Bristol Myers Squibb withdrew the initial FDA approval for nivolumab.

Although a randomized phase III trial of first-line therapy comparing chemotherapy versus chemotherapy plus pembrolizumab (Keynote 604) did not reach the statistical threshold for improvement in overall survival [7], two other recent large, randomized phase III trials (Impower 133 and CASPIAN) demonstrated that the addition of a monoclonal antibody targeting programmed death ligand-1 (anti-PD-L1) (atezolizumab or durvalumab), in combination with platinum plus etoposide, led to a significant, but modest, improvement in progression-free survival (PFS) and overall survival (OS), prompting FDA approval. This has changed current practice. As of 2024, combination regimens of immunotherapy (anti-PD-L1 antibodies) plus chemotherapy are the standard of care in the first-line treatment of extensive-stage SCLC [8,9,10,11].

Despite the marginal improvement in survival provided by the combination of anti-PD-L1 monoclonal antibodies and chemotherapy, a subset of patients derived long-term benefit [9,12,13]. Although the survival gains in this unselected population were modest (~2 months), a subset of patients achieved significant clinic benefit, with 16–18% exhibiting a durable response at 3 years [9,12,13]. The abovementioned Keynote 604 trial also had a small percentage of patients with long-term survival. These results highlight the need to develop biomarkers to better identify the subgroup of patients most likely to benefit from immunotherapy and further understand mechanisms of resistance to immunotherapeutic approaches so that improved combination strategies may be developed. The purpose of this study was to perform whole-transcriptome sequencing of tumors from a cohort of extensive-stage SCLC treated with ICB, outside of a clinical trial setting, in both the first-line and second-line settings to assess and compare the association of specific transcriptional signatures with responses to ICB.

The genetic profiling of SCLC typically reveals a high tumor mutation burden with the frequent inactivation of the tumor suppressor genes *TP53* and *RB1* [14]. As opposed to non-small-cell lung cancer (NSCLC), where the identification of oncogenic driver mutations and expression levels of PD-L1 is utilized to guide therapeutic decision making, the genetic profiling of SCLC has not identified specific mutational subtypes and PD-L1 expression has not been proven to be a predictive biomarker [2,13,15]. 

However, the transcriptional profiling of SCLC does appear to define molecularly distinct subgroups identified by the relative expression of the transcriptional factors ASCL1, NEUROD1, POU2F3, and YAP1 [16,17,18,19]. In addition to defining molecular subtypes, the RNA sequencing of SCLC tumors offers an opportunity to interrogate multiple specific transcriptional programs and elucidate potential therapeutic vulnerabilities within the tumor microenvironment. For instance, the T cell-inflamed gene expression profile (GEP) signature is associated with responses to checkpoint blockade in the relapsed setting in a variety of tumor types, including SCLC [5,20,21]. The activation of Notch signaling has also been associated with responses to checkpoint blockade in second-line patients [22]. However, determining whether these signatures are predictive in the first-line setting using combination therapy (the current standard of care) remains understudied and was an aim of this investigation. 

## 2. Materials and Methods

### 2.1. Study Design and RNA Sequencing

This single-center, retrospective, and observational study was conducted at the Hospital of the University of Pennsylvania from October 2021 to December 2023 and was approved by the University’s Institutional Review Board. The Strengthening the Reporting of Observational Studies in Epidemiology (STROBE) statement was followed to ensure the quality of the data reported in this study [23]. 

The SCLC cohort was composed of patients with extensive-stage SCLC treated with anti-PD1 or anti-PD-L1 antibodies who had sufficient residual formalin-fixed paraffin-embedded (FFPE) tumor material for detailed RNA analyses [24]. A thoracic pathologist confirmed the presence of adequate tumor material in FFPE slides. Initially, 66 subjects were identified, of whom 40 samples were submitted for RNA extraction, and 35 had RNA of sufficient quality for analysis. Baseline demographics and clinical variables were obtained from the electronic medical record. 

RNA was analyzed on an HTG EdgeSeq Processor (HTG Molecular Diagnostic, Tucson, AZ, USA) using an HTG Transcriptome Panel (targeting >20,000 genes), as described previously [25,26]. Briefly, functional nuclease protection probes were hybridized to target RNA. S1 nuclease was then added to digest non-hybridized probes and RNA, leaving only the nuclease protection probes hybridized to their respective targets. Heat denaturation was utilized to release the DNA:RNA duplexes and the DNA protection probes were amplified and barcoded to generate sequencing libraries. Libraries were sequenced on the Illumina NextSeq 500/550 platform (Illumina, San Diego, CA, USA) according to the manufacturer’s instructions. Gene expression data were imported into the 2022 HTG EdgeSeq Parser software (https://www.htgmolecular.com/resources/view/htg-edgeseq-workflow (accessed on 28 July 2024)) for alignment of FASTQ files to the probe list and quantification of the reads. The HTG EdgeSeq Reveal application was utilized to quality check and normalize the data. The data were normalized using counts per million reads and log2-transformed. 

### 2.2. Gene Signature Generation

The goal of this study was to evaluate a number of transcriptional signatures and their association with responses to ICB in SCLC. Log2 z-score gene signatures reflecting inflammation, the epithelial–mesenchymal transformation (EMT), and antigen-processing machinery (APM) were generated as previously described [5,20,24,27]. 

Based on the publication of Zhang et al. [28], we generated a neuroendocrine (NE) score for each tumor by calculating a log2 z-score for high-NE genes and subtracting the z-scores of low-NE genes (Supplemental Table S1). The genes used for each signature are summarized in Supplemental Table S1. 

### 2.3. Immunohistochemistry

An additional 4 patients had biopsy samples in which tissue was adequate for immunohistochemical staining. The final group of 39 patients included 20 first-line patients (11 with no durable clinical benefit (NDB) and 9 with durable clinical benefit (DCB)) and 19 second-line patients (13 with NDB and 6 with DCB). FFPE sections were stained with INSM1 [A-8, Santa Cruz, sc-271408], CD8 antibodies [C8/144B, Cell Marque, 108M-96], visualized with Opal dyes, and counterstained with DAPI. The slides were digitally scanned on Vectra Polaris at 20× (Akoya, Marlborough, MA, USA). Evaluable tissue areas were annotated by a pathologist (MK) and then segmented into tumor versus stroma based on the expression of INSM1. CD8 T cells were counted using Visiopharm software version 2023.01 [Hørsholm, Denmark] and normalized to their respective tissue areas. 

### 2.4. Statistical Analyses of Cohort

Descriptive statistics were computed for patients and treatment characteristics. Response variables were grouped into the binary categories of patients having a durable clinical benefit (DCB) from therapy (progression-free survival ≥ 6 months) or patients with no durable benefit (NDB) to therapy (progression-free survival < 6 months). The association between a gene signature score and clinical benefit was examined using a *t*-test after the normality of the gene scores was established. Associations between gene signatures were examined using Pearson correlation coefficients for continuous variables. All statistical analyses were two-sided and performed using Graphpad Prism version 10.0 (La Jolla, CA, USA).

## 3. Results

### 3.1. Patient Characteristics

We obtained archival pre-treatment tumor specimens from a cohort of 66 extensive SCLC patients treated with immune checkpoint blockade at our institution. Thirty-five subjects had sufficient material for whole-transcriptome sequencing. Baseline characteristics are provided in Supplemental Table S2. The median age was 67 years, 60% were female, and 97% were current or former smokers. Eleven patients (31%) had durable clinical benefit (DCB) (response lasting ≥ 6 months) with a median overall survival for the entire cohort of 6.6 months (Supplementary Figure S1). 

Twenty-one patients were treated with ICB in the first-line setting with atezolizumab plus platinum–etoposide every 3 weeks. Eight had DCB and thirteen had no DCB. The mean progression-free survival (PFS) was 31 weeks and the mean overall survival (OS) was 46 weeks. Fourteen patients were treated in the relapsed setting with ICB. Eleven patients received nivolumab, two patients received both ipilumimab and nivolumab, and one patient received atezolizumab. Three patients had DCB and eleven had no DCB. The mean PFS was 21 weeks, and the mean OS was 32 weeks (Supplementary Figure S1). PFS and OS were not significantly different in the first- versus second-line patients (*p* = 0.19 and *p* = 0.21, respectively).

### 3.2. Comparison to Other SCLC Genomic Datasets with Regard to Transcriptional Subtypes

Because our cohort was relatively small and we employed probe-based transcriptomic profiling technology (HTG EdgeSeq—see Section 2.1) as opposed to conventional RNA sequencing methods, we verified that our genomic information evaluating the transcriptional subclassifications of SCLC and their relationships to key pathways was similar to previously published data. 

#### 3.2.1. Transcriptional Subtypes

**Figure 1:**
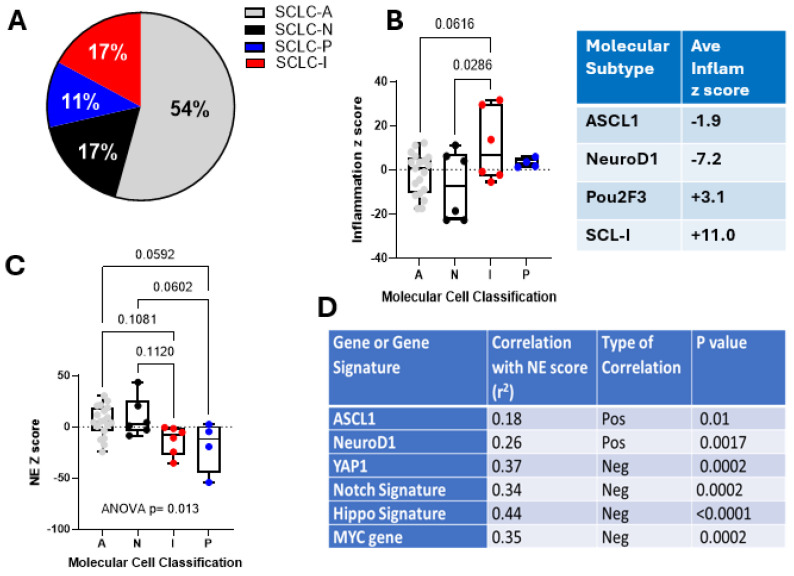
Molecular subtypes in patient cohort. (**A**) The molecular subtype of each tumor. SCLC, including ASCL1 (SCLC-A), NEUROD1 (SCLC-N), POU2F3 (SCLC-P), and the SCLC-I subtype, was defined by transcriptomic analysis. (**B**) The inflammation log2 z-score was determined for each subtype. The SCLC-I subtype demonstrated the upregulation of the inflammatory gene signature. (**C**) The neuroendocrine (NE) log2 z score was determined for each subtype. (**D**) The table shows the correlation of expression of the ASCL1, NeuroD1, YAP1, and MYC genes, as well as the NOTCH and HIPPO gene signatures with the NE log2 gene score. The type of correlation (positive or negative) and the *p* values are listed. Multiple comparisons were made using ANOVA with post hoc testing. Pearson correlations and *p* values are shown.

We first evaluated the differential expression of transcripts previously reported to define distinct molecular subtypes of SCLC, including *ASCL1* (SCLC-A), *NEUROD1* (SCLC-N), *POU2F3* (SCLC-P)*,* and the SCLC-I subtype (defined as having low-level expression of these transcripts, with high *YAP1*) (Figure 1A, Supplementary Figure S2). The most common subtype was SCLC-A, encompassing 54% of the cohort, followed by SCLC-N and SCLC-I, both representing 17%, with SCLC-P accounting for 11%. This subtype distribution was very similar to the previously reported analysis of the IMpower133 dataset (SCLC-A 51%, SCLC-N 23%, SCLC-I 18%, and SCLC-P 7%) [19]. The SCLC-I subtype, which exhibits a more inflammatory phenotype [19], demonstrated upregulation of the inflammatory gene signature (Figure 1B). 

#### 3.2.2. Genes and Pathways Affecting Transcriptional Subtypes

Previous reports have shown that the neuroendocrine subtypes, SCLC-A and SCLC-N, demonstrate greater neuroendocrine differentiation [19,29] and that neuroendocrine differentiation was inversely correlated with the *NOTCH*, *HIPPO*, and *MYC* signaling pathways [19,22,28,30]. Congruent with these reports, we observed that, in our cohort, the NE scores were higher in the ASCL1 and NEUROD1 tumors (Figure 1C), and that the neuroendocrine (NE) score was positively correlated with expression levels of *ASCL1* (Supplementary Figure S3A) and *NEUROD1* (Supplementary Figure S3B). The NE score was inversely correlated with the expression of *YAP1* (Supplementary Figure S3C), *MYC* (Supplementary Figure S3D), a *NOTCH* signaling signature (Supplementary Figure S3E), and a *HIPPO* signaling signature (Supplementary Figure S3F), as summarized in Figure 1D.

*YAP1* expression has been associated with a T cell-inflamed phenotype [31]. In our data, we also observed a strong correlation of *YAP1* with an inflammation score (Supplementary Figure S4A), a T cell score (Supplementary Figure S4B), and an antigen presentation machinery score (Supplementary Figure S4C). Similarly, we also noted a strong correlation of the *HIPPO* signaling score with inflammation (Supplementary Figure S4D).

In summary, these data show that the transcriptomic data from our cohort were highly congruent with those published in the literature, supporting further analyses.

### 3.3. Gene Signatures and Responses to ICB

Given this validation, we next sought to determine whether specific gene signatures demonstrated to predict responses to ICB in NSCLC, and in a variety of other tumor types, would be associated with responses to ICB in our cohort of SCLC patients. 

#### 3.3.1. Overall Cohort

**Figure 2:**
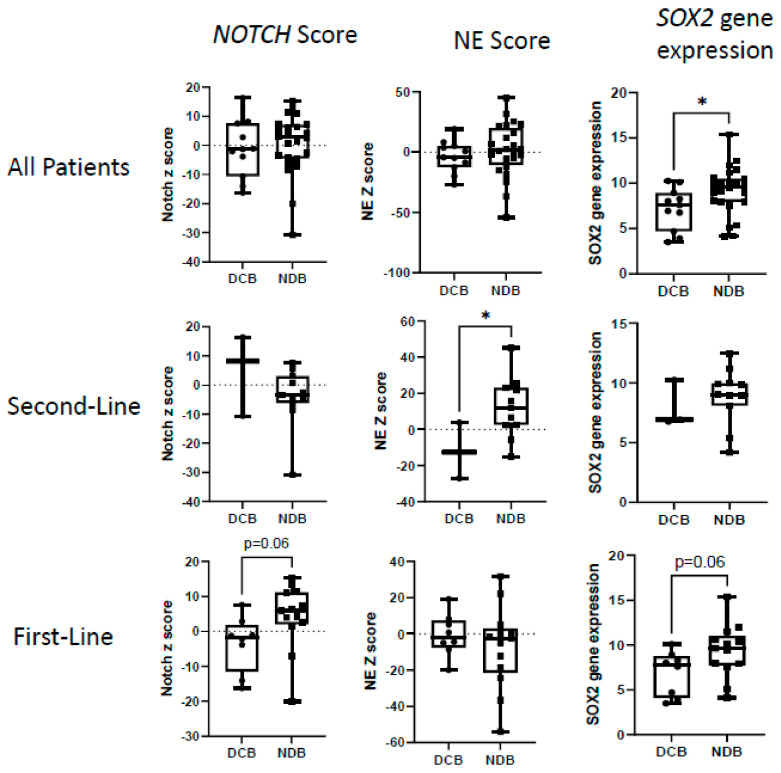
Relationship of key pathway signatures to clinical responses. The log2 z-score for the *NOTCH* pathway, neuroendocrine (NE) signature, and *SOX2* gene expression are plotted for patients with durable clinical benefit (DCB) or no durable clinical benefit (NDB). The upper panels show data from the entire cohort of patients. The middle panels show data from the second-line patients. The lower panels show data from the first-line patients. Values were compared using *t* tests. * = *p* < 0.05.

Previous transcriptomic studies on SCLC have reported the following: (1) elevated *NOTCH* signaling has been associated with responses to ICB [22], (2) patients with lower NE scores had a better response to ICB [32] and (3) the upregulation of *SOX2* mediates the exclusion of CD8+ T cells from the tumor microenvironment and may play a role in mediating resistance to checkpoint blockade [33,34]. We examined these oncogenic pathways in the overall cohort, comparing those patients with durable clinical benefit (DCB) to those with no durable benefit (NDB) (Figure 2, upper panels). No significant differences in the *NOTCH* or NE scores in relationship to responses were observed; however, the expression of *SOX2* was significantly higher in those patients with NDB (Figure 2, upper panels). No significant differences were observed in the *HIPPO* signaling score, the *HEDGEHOG* gene score, the *HEDGEHOG* signaling score, nor the *MYC* gene expression levels between patients with DCB and NCB (Supplementary Figure S5, upper panels). 

**Figure 3:**
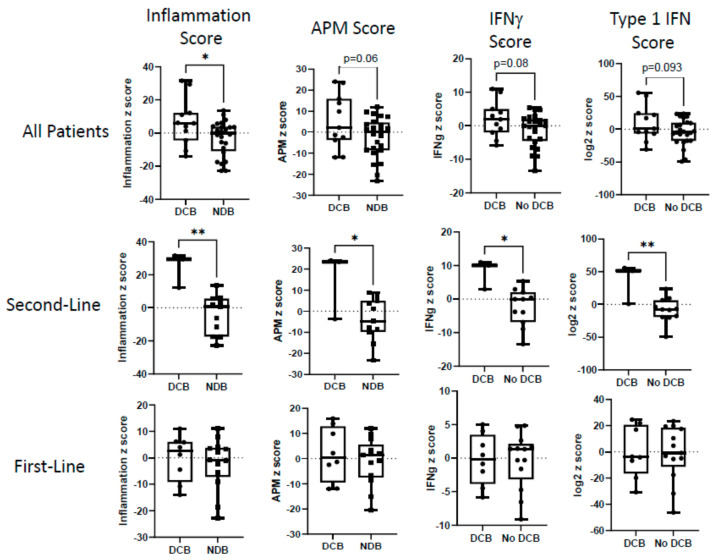
Relationship of inflammatory gene signatures to clinical responses. The log2 z-scores for the inflammation pathway, antigen presentation machinery (APM), interferon-g pathway, and type 1 interferon pathway are plotted for patients with durable clinical benefit (DCB) or no durable clinical benefit (NDB). The upper panels show data from the entire cohort of patients. The middle panels show data from the second-line patients. The lower panels show data from the first-line patients. Values were compared using *t* tests. * = *p* < 0.05, ** = *p* < 0.01.

**Figure 4:**
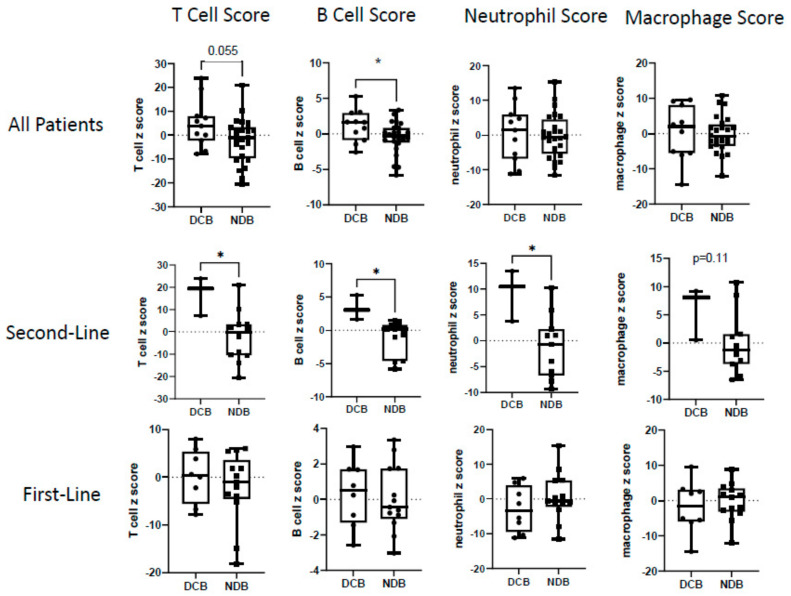
Relationship of cell-type gene signatures to clinical responses. The log2 z-scores for T cells, B cells, neutrophils, and macrophages are plotted for patients with durable clinical benefit (DCB) or no durable clinical benefit (NDB). The upper panels show data from the entire cohort of patients. The middle panels show data from the second-line patients. The lower panels show data from the first-line patients. Values were compared using *t* tests. * = *p* < 0.05.

We next examined an inflammatory gene signature, a signature representing antigen presentation machinery (APM) genes, an interferon-γ score, and a type 1 interferon score (Figure 3, upper panels), as well as specific immune cell signatures (Figure 4, upper panels). The inflammation gene signature and the B cell score were significantly (*p* < 0.05) higher in the DCB patients. The APM score (*p* = 0.06), interferon-γ score (*p* = 0.08), type 1 interferon score (*p* = 0.09), and T cell score (*p* = 0.055) showed strong trends toward significance compared to the non-responders. The neutrophil score and macrophage score were not significantly different between the DCB and NDB groups.

#### 3.3.2. First-Line vs. Second-Line Patients

The vast majority of studies performed to date (including our own studies with NSCLC [24,27]) demonstrating the ability of gene signatures to predict responses to checkpoint blockade have focused on patients treated with ICB alone in second-line or relapsed settings [5,20,22,35]. However, the current standard of care for small-cell lung cancer is to use ICB in combination with chemotherapy in the first-line setting [11]. Accordingly, we reanalyzed our data by separately grouping those patients who were treated in the second-line setting with ICB therapy after relapse versus the first-line patients treated with chemotherapy plus ICB.

In these analyses, we noted marked differences in predictive gene signatures between the second- and first-line patients (middle panels versus lower panels in Figure 2, Figure 3 and Figure 4). Even though our cohort of second-line patients was small (n = 14, with 3 patients having DCB and 11 patients with NDB), our results were statistically significant and very similar to those described in second-line SCLC [22,35] as well as most other tumors [5], in that inflamed tumors were significantly more responsive to checkpoint blockade. 

##### Second-Line Patients

The second-line patients with DCB compared to NDB showed significantly higher values in their inflammation scores (average z-score: 24.4 vs. −4.0; *p* = 0.003), APM scores (average z-score: 14.6 vs. −3.9; *p* = 0.03), IFN-γ scores (average z-score: 8.0 vs. −2.4; *p* = 0.012), and Type 1 IFN (average z-score: 36.1 vs. −8.4; *p* = 0.008) scores at baseline (Figure 3, middle panels). Very strong differences in expression were noted in many of the individual genes in these signatures, especially those that overlapped, such as *IDO1*, *PD-L1*, *CXCL9*, and *CCL5* (Supplementary Figure S5). The second-line patients with DCB also had significantly higher T cell scores (average z-score: 16.8 vs. −2.0; *p* = 0.02), B cell scores (average z-score: 3.3 vs. −1.2; *p* = 0.016), neutrophil scores (average z-score: 9.2 vs. −1.3; *p* = 0.019), and macrophage scores (average z-score: 5.9 vs. −0.10; *p* = 0.11) (Figure 4, middle panels). Patients with lower NE scores were significantly more likely to respond (average z-score: −10.8 vs. 13.1; *p* = 0.04) (Figure 2, middle panels).

##### First-Line Patients

First-line patients treated with chemotherapy and ICB (n = 21, with 8 patients having DCB and 13 with NDB) showed a completely different pattern; tumor inflammation status yielded no predictive pattern (Figure 3 and Figure 4, lower panels). In contrast to the second-line patients, there were no significant differences in inflammation (average z-score: −0.12 vs. −2.2, *p* = 0.64), APM (average z-score: 1.8 vs. −1.1, *p* = 0.52), IFN-γ (average z-score: −0.21 vs. −0.35, *p*= 0.94), Type 1 IFN (average z-score: −0.2 vs. −1.1, *p* = 0.92), T cell (average z-score: 0.2 vs. −2.1, *p* = 0.46), B cell (average z-score: 0.31 vs. 0.06, *p* = 0.77), neutrophil (average z-score: −2.9 vs. 0.7, *p* = 0.25), and macrophage scores (average z-score: −1.7 vs. −0.2; *p* = 0.60). NE scores were not significantly different; however, there were strong trends favoring better responses in patients with lower *NOTCH* scores (average z-score: −3.9 vs. 3.9, *p* = 0.06) and lower *SOX2* gene expression (average z-score: 6.9 vs. 9.3, *p* = 0.06) (Figure 2, lower panels). 

We saw no differences in the *HIPPO* signaling scores, the *HEDGEHOG* gene scores, *HEDGEHOG* signaling scores, nor *MYC* gene expression levels between patients with DCB and NCB in either the first or second line (Supplementary Figure S6). 

#### 3.3.3. Immunohistochemical Validation

**Figure 5:**
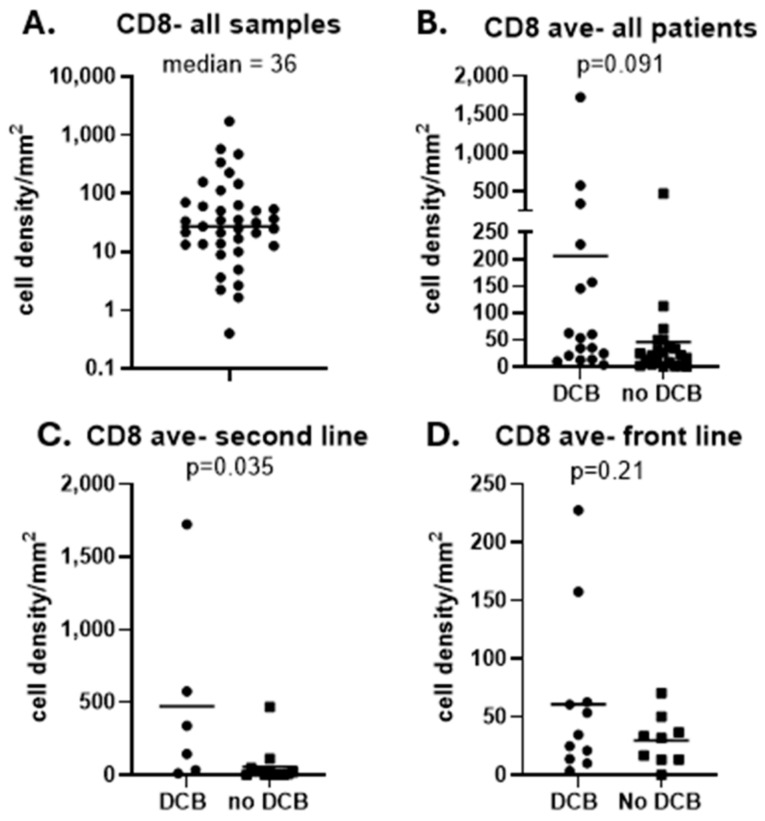
Relationship of CD8 T cell immunohistochemistry to clinical responses. (**A**). The cell density (cells/mm^2^ of tumor) of CD8 T cells determined by immunohistochemistry is plotted (on a logarithmic scale) for all 36 patients. The median value is 36 cells/ mm^2^. The cell densities of CD8 T cells are plotted for patients with durable clinical benefit (DCB) or no durable clinical benefit (NDB). Panel (**B**) shows data from the entire cohort of patients. Panel (**C**) shows data from the second-line patients. Panel (**D**) shows data from the first-line patients. Values were compared using *t* tests. Exact *p* values are shown.

To validate our genomic findings showing that T cell infiltration was correlated with responses to ICB in only the second-line patients, we stained 39 patient samples (including the 35 used for mRNA analysis plus 4 additional patients whose RNA quality was not adequate) for INSM1 to mark SCLC tumor cells and CD8 for cytotoxic T cells on the same section (Supplementary Figure S7). Consistent with previous studies on SCLC [36], the density of CD8+ T cells was quite low (median: 36 cells/mm^2^), but with a large spread of values (Figure 5A) compared to other tumors [37]. There was a trend towards a higher number of CD8+ T cells in the overall group of patients with DCB (Figure 5B; *p* = 0.09); however, this was driven mostly by the second-line patients (Figure 5C; *p* = 0.035) compared to the first-line patients (Figure 5D; *p* = 0.21). These data are highly congruent with the genomic T cell scores (Figure 4), showing that the presence of higher numbers of CD8+ T cells predicted responses in the second-line, but not front-line patients.

## 4. Discussion

Although the addition of immunotherapy to platinum-based chemotherapy has emerged as a new standard of care for patients with extensive-stage SCLC, the overall benefit of this approach in an unselected patient population remains modest [4,9,11,13]. Only a subset of patients demonstrate durable clinical responses [9,12]. In addition, novel therapeutic strategies for SCLC are rapidly emerging [38,39], which further underscores the need to identify predictive biomarkers to tailor treatment to different subtypes of SCLC. 

However, there is a current knowledge gap as data describing predictive biomarkers for ICB are almost exclusively derived from earlier trials examining patients treated in the second-line setting. For example, the recent study by Rudin et al. [35] analyzed data from the CheckMate 032 trial, a second-line therapy trial in which SCLC patients whose disease had progressed on one or more platinum-based chemotherapies were treated with nivolumab monotherapy or nivolumab combined with ipilimumab. They found that genes associated with antigen processing and presentation were enriched in patients with a durable clinical benefit [35] and that the number of infiltrating CD8 T cells (by immunohistochemistry) correlated with survival in patients treated with nivolumab. They also observed that inflammation and interferon signatures correlated with responses [35] (p 1228, Fig. 4B). 

The primary goal of our study was to extend this biomarker exploration to SCLC patients treated in the first-line setting with combination chemotherapy and ICB. Since our study cohort was relatively small and we used a different transcriptomic analysis technique compared to previous studies, we first compared our data to those of previous studies. We found that our findings were highly consistent with published studies in terms of molecular subtype classification frequency, the relationships of molecular subtypes with known oncogenic pathways, and the relationships of neuroendocrine statuses with signaling pathways, as well as inflammation signatures.

Furthermore, when we analyzed our group of patients treated in the second line with ICB alone, our data were also very similar to the previously described predictive relationships between the underlying inflammatory states of tumors and responses to ICB that have been noted in many tumor types [5,20,24,27] and by Rudin et al. in second-line ICB-treated SCLC [35]. Despite having only a small number of patient samples, we found that the second-line SCLC patients with durable responses had statistically higher transcriptomic levels of genes associated with inflammation, antigen presentation machinery, and interferon responses. In addition, a T cell signature was increased, and significantly more CD8+ T cells were present in the tumor microenvironment by immunostaining. In these tumors, an anti-tumor immune response appears to have been initiated, but then inhibited (presumably by upregulation of T cell inhibitory receptors like PD1 and CTLA-4), that was then “unleashed” by checkpoint blockade.

Given these multiple validations of our dataset, we felt confident in studying biomarkers in our first-line SCLC patients treated with chemotherapy plus ICB. Somewhat surprisingly, we found that, in contrast to the second-line patients, none of these indicators of an activated tumor microenvironment, nor the presence of increased T cells, predicted responses to ICB in the first-line setting where chemotherapy was also given.

The reason for this dichotomy is unknown, but one possible explanation is that chemotherapy is able to alter the tumor cells and/or tumor microenvironment towards a more immunogenic state that could then be amplified by the presence of anti-PD-(L)1 antibodies [11]. There is a solid body of literature describing how platinum agents (including carboplatin and cisplatin) can have significant immunomodulatory effects [40,41,42,43,44]. The proposed mechanisms include the following: (1) the upregulation of MHC class 1 expression on tumor cells (allowing enhanced antigen presentation and recognition by cytotoxic T cells), (2) the recruitment of effector immune cells like CD8+ T cells and dendritic cells, (3) the increased lytic activity of cytotoxic T cells and NK cells, (4) the downregulation of immunosuppressive factors in the tumor microenvironment, and (5) the induction of immunogenic cell death, leading to danger signals and the release of tumor antigens. The upregulation of MHC class 1 expression might be especially important in SCLC, since most tumors show marked downregulation of MHC class 1 and beta2 microglobulin, making them relatively “invisible” to cytotoxic T cells [35,45,46,47]. Because of these multiple stimulatory effects, we postulate that an underlying “hot tumor” phenotype is not required for efficacy when ICB is combined with chemotherapy.

Although we were easily able to find many transcriptomic predictors of responses to ICB in our second-line patients, predictors of responses to first-line combined therapies were more difficult to identify. A higher expression of the *NOTCH* pathway signature exhibited a strong trend (*p* = 0.06) as a negative predictive factor for responses. A higher expression of the transcription factor *SOX2* was associated with poor responses in all groups of patients, but a high expression of *SOX2* had previously been associated with a poor prognosis in SCLC before the use of ICB [48].

*NOTCH* pathway genes were of special interest. Roper et al. [22] found an increase in *NOTCH* pathway genes associated with clinical benefit after treatment with ICB in second-line patients. Our data are consistent with this observation (Figure 2); however, in our small dataset this difference did not reach significance. In contrast, the opposite association was seen in our first-line patients, where an increase in Notch pathway genes was associated with lack of clinical benefit. Further research with additional patients will be needed to validate this association, but *NOTCH*-1 inhibitors are being studied and could potentially be of use in first-line patients [49].

During the final preparation of this manuscript, an additional analysis of the first-line IMpower 133 study, using 271 patient samples, was published [50]. The authors applied non-negative matrix factorization to refine cellular subtypes of SCLC and found that there were two inflamed subsets with different clinical outcomes dependent on the ratio of T-effector to tumor-associated macrophage infiltration; inflammatory tumors with high CD8 T cells and low macrophages (~14% of their population) showed enhanced benefit. We looked for a similar group of patients in our cohort, but found that there was a very high positive correlation between CD8 T cell z-score and macrophage z-score and were thus unable to identify any patients with a high CD8 to macrophage z-score ratio. The small size of our dataset could certainly limit our ability to detect smaller effect size predictors.

This study has a number of limitations that should be considered. Our biopsies were often small core needle or bronchoscopic samples rather than surgical specimens, so tumor heterogeneity might have affected our results and induced some variability. The biopsies were taken at the time of initial diagnosis and were thus obtained close to when first-line therapy was administered. Therefore, we did not have repeat biopsies when second-line immune checkpoint blockade (ICB) therapy was initiated. Since initial biopsies that were inflammatory were highly predictive of responses after chemotherapy failed, we speculate that the tumor “hotness setpoint” is intrinsic and that changes induced by chemotherapy were relatively transitory, with the tumor returning to its natural setpoint. This setpoint is likely defined by specific tumor-intrinsic characteristics, such as mutational burden, differentiation status, the activation of key oncogenic pathways (such as *NOTCH* and *HIPPO*), the degree of the epigenetic downregulation of HLA class 1 and antigen-presenting machinery, and specific chemokine production. It would thus be instructive to determine how chemotherapy affected the tumor microenvironment; however, at this time, repeat biopsies at the time of disease progression are not the standard of care.

Another important limitation is that the study was performed at a single institution and the number of patients studied (n = 35) was relatively small. Although we tried to select for patients with responses, only three of fourteen patients had durable clinical responses in our second-line cohort, markedly limiting our statistical power. Despite this, many associations in this group were still statistically significant, suggesting strong effects. Our first-line patient group had more patients and a better balance of responders (n = 8) and non-responders (n = 13); however, the lack of significant associations in our first-line patients should be tempered by our relatively low level of power. Our findings should thus be considered hypothesis-generating. It would be important for these results to be replicated in larger and more varied groups of SCLC patients.

With these caveats in mind, there are a number of possible implications of our study. Our data confirmed that relatively few SCLC tumors were inflammatory; however, if an SCLC appears to have an intrinsic “hot tumor” set point, it might be advantageous to re-challenge such patients with some variation in ICB. For example, one could institute an anti-PD-1 antibody, an anti-CTLA4 antibody, or one of the newly developing types of checkpoint blockade. Combining these antibodies with LSD1 inhibitors might be particularly helpful, as recent data suggest that the epigenetic modifier LSD1 might upregulate MHC class 1 and enhance antigen presentation as well as T cell responses [51,52,53]. In contrast, our data suggest that LSD1 inhibitors might not be necessary in first-line patients treated with combination therapies, as the chemotherapy might be having similar immune-activating effects. Our data also suggest that a relatively simple and inexpensive test, such as immunostaining for CD8 T cell density, could be useful in classifying a tumor as “hot” and thus more likely to respond to immunotherapy alone.

## 5. Conclusions

In summary, our data suggest that the response to ICB in SCLC in the second-line setting was predicted by the baseline inflammatory state of the tumor. In contrast, this strong association with inflammation was not observed in the first-line setting where chemotherapy was combined with anti-PDL1 blockade. No clear biomarkers of responses in the first-line setting were identified; however, patients with durable clinical benefit had lower expression of Notch pathway genes and SOX2 gene expression. A better understanding of how the addition of chemotherapy to ICB affects responses might help to better identify potential responders and to help augment or prolong responses.

## Data Availability

The datasets generated and analyzed during the current study are available from the corresponding author upon reasonable request.

## Supplementary Materials

The following supporting information can be downloaded at: https://www.mdpi.com/article/10.3390/cancers16162795/s1, Table S1. Gene Signatures [20,22,24,27,28,30,54]. Table S2. Clinical Characteristics of the Patients. Figure S1. Characterization of Patients; Figure S2. SLCL Subtype Classifications; Figure S3. Correlations of combined neuroendocrine log2 z scores with key pathways; Figure S4. Correlations of YAP1 gene count with inflammation, T cell, and antigen presentation machinery log2 z scores; Figure S5. IDO1, CXCL9, and CCL5 scores for patients with DCB vs NDB; Figure S6. HIPPO, HEDGEHOG, and MYC scores for patients with DCB vs NDB; Figure S7. CD8 T cell staining.

## References

[1] NCCN (2023). NCCN Guidelines Version 1.2024: Small Cell Lung Cancer. http://www.nccn.org.

[2] Rudin C.M., Brambilla E., Faivre-Finn C., Sage J. (2021). Small-cell lung cancer. Nat. Rev. Dis. Primers.

[3] Antonia S.J., Lopez-Martin J.A., Bendell J., Ott P.A., Taylor M., Eder J.P., Jager D., Pietanza M.C., Le D.T., de Braud F. (2016). Nivolumab alone and nivolumab plus ipilimumab in recurrent small-cell lung cancer (CheckMate 032): A multicentre, open-label, phase 1/2 trial. Lancet Oncol..

[4] Li L., Liang Y., Yu M., Zhao L., Mei Q., Yu Y., Wang N., Zhang D., Wang Z., Jia Y. (2023). Advances in immune checkpoint inhibitors therapy for small cell lung cancer. Cancer Med..

[5] Ott P.A., Bang Y.J., Piha-Paul S.A., Razak A.R.A., Bennouna J., Soria J.C., Rugo H.S., Cohen R.B., O’Neil B.H., Mehnert J.M. (2019). T-cell-inflamed gene-expression profile, programmed death ligand 1 expression, and tumor mutational burden predict efficacy in patients treated with pembrolizumab across 20 cancers: KEYNOTE-28. J. Clin. Oncol..

[6] Ready N.E., Ott P.A., Hellmann M.D., Zugazagoitia J., Hann C.L., de Braud F., Antonia S.J., Ascierto P.A., Moreno V., Atmaca A. (2020). Nivolumab monotherapy and nivolumab plus ipilimumab in recurrent small cell lung cancer: Results from the checkMate 032 randomized cohort. J. Thorac. Oncol..

[7] Rudin C.M., Awad M.M., Navarro A., Gottfried M., Peters S., Csoszi T., Cheema P.K., Rodriguez-Abreu D., Wollner M., Yang J.C. (2020). Pembrolizumab or placebo plus etoposide and platinum as first-line therapy for extensive-stage small-cell lung cancer: Randomized, double-blind, phase III KEYNOTE-604 study. J. Clin. Oncol..

[8] Horn L., Mansfield A.S., Szczesna A., Havel L., Krzakowski M., Hochmair M.J., Huemer F., Losonczy G., Johnson M.L., Nishio M. (2018). First-line atezolizumab plus chemotherapy in extensive-stage small-cell lung cancer. N. Engl. J. Med..

[9] Paz-Ares L., Chen Y., Reinmuth N., Hotta K., Trukhin D., Statsenko G., Hochmair M.J., Özgüroglu M., Ji J.H., Garassino M.C. (2022). Durvalumab, with or without tremelimumab, plus platinum-etoposide in first-line treatment of extensive-stage small-cell lung cancer: 3-year overall survival update from CASPIAN. Esmo Open.

[10] Paz-Ares L., Dvorkin M., Chen Y., Reinmuth N., Hotta K., Trukhin D., Statsenko G., Hochmair M.J., Ozguroglu M., Ji J.H. (2019). Durvalumab plus platinum-etoposide versus platinum-etoposide in first-line treatment of extensive-stage small-cell lung cancer (CASPIAN): A randomised, controlled, open-label, phase 3 trial. Lancet.

[11] Zhang S., Cheng Y. (2023). Immunotherapy for extensive-stage small-cell lung cancer: Current landscape and future perspectives. Front. Oncol..

[12] Liu S.V., Dziadziuszko R., Sugawara S., Kao S., Hochmair M., Huemer F., de Castro G., Havel L., Caro R.B., Losonczy G. Five-year survival in patients with ES-SCLC treated with atezolizumab in IMpower133: IMbrellla A extension study results. Proceedings of the 2023 World Conference on Lung Cancer.

[13] Liu S.V., Reck M., Mansfield A.S., Mok T., Scherpereel A., Reinmuth N., Garassino M.C., De Castro Carpeno J., Califano R., Nishio M. (2021). Updated overall survival and PD-L1 subgroup analysis of patients with extensive-stage small-cell lung cancer treated with atezolizumab, carboplatin, and etoposide (IMpower133). J. Clin. Oncol..

[14] George J., Lim J.S., Jang S.J., Cun Y., Ozretic L., Kong G., Leenders F., Lu X., Fernandez-Cuesta L., Bosco G. (2015). Comprehensive genomic profiles of small cell lung cancer. Nature.

[15] Paz-Ares L., Goldman J.W., Garassino M.C., Dvorkin M., Trukhin D., Statsenko G., Hotta K., Ji J.H., Hochmair M.J., Voitko O. (2019). PD-L1 expression, patterns of progression and patient-reported outcomes (PROs) with durvalumab plus platinum-etoposide in ES-SCLC: Results from CASPIAN. Ann. Oncol..

[16] Borromeo M.D., Savage T.K., Kollipara R.K., He M., Augustyn A., Osborne J.K., Girard L., Minna J.D., Gazdar A.F., Cobb M.H. (2016). ASCL1 and NEUROD1 reveal heterogeneity in pulmonary neuroendocrine tumors and regulate distinct genetic programs. Cell Rep..

[17] Huang Y.H., Klingbeil O., He X.Y., Wu X.S., Arun G., Lu B., Somerville T.D.D., Milazzo J.P., Wilkinson J.E., Demerdash O.E. (2018). POU2F3 is a master regulator of a tuft cell-like variant of small cell lung cancer. Genes Dev..

[18] Rudin C.M., Poirier J.T., Byers L.A., Dive C., Dowlati A., George J., Heymach J.V., Johnson J.E., Lehman J.M., MacPherson D. (2019). Molecular subtypes of small cell lung cancer: A synthesis of human and mouse model data. Nat. Rev. Cancer.

[19] Gay C.M., Stewart C.A., Park E.M., Diao L., Groves S.M., Heeke S., Nabet B.Y., Fujimoto J., Solis L.M., Lu W. (2021). Patterns of transcription factor programs and immune pathway activation define four major subtypes of SCLC with distinct therapeutic vulnerabilities. Cancer Cell.

[20] Ayers M., Lunceford J., Nebozhyn M., Murphy E., Loboda A., Kaufman D.R., Albright A., Cheng J.D., Kang S.P., Shankaran V. (2017). IFN-gamma-related mRNA profile predicts clinical response to PD-1 blockade. J. Clin. Investig..

[21] Cristescu R., Mogg R., Ayers M., Albright A., Murphy E., Yearley J., Sher X., Liu X.Q., Lu H., Nebozhyn M. (2018). Pan-tumor genomic biomarkers for PD-1 checkpoint blockade-based immunotherapy. Science.

[22] Roper N., Velez M.J., Chiappori A., Kim Y.S., Wei J.S., Sindiri S., Takahashi N., Mulford D., Kumar S., Ylaya K. (2021). Notch signaling and efficacy of PD-1/PD-L1 blockade in relapsed small cell lung cancer. Nat. Commun..

[23] von Elm E., Altman D.G., Egger M., Pocock S.J., Gotzsche P.C., Vandenbroucke J.P., Initiative S. (2008). The strengthening the reporting of observational studies in epidemiology (STROBE) statement: Guidelines for reporting observational studies. J. Clin. Epidemiol..

[24] Thompson J.C., Hwang W.T., Davis C., Deshpande C., Jeffries S., Rajpurohit Y., Krishna V., Smirnov D., Verona R., Lorenzi M.V. (2020). Gene signatures of tumor inflammation and epithelial-to-mesenchymal transition (EMT) predict responses to immune checkpoint blockade in lung cancer with high accuracy. Lung Cancer.

[25] Borchert S., Herold T., Kalbourtzis S., Hamacher R., Krause Y., Berger S., Guder W.K., Streitbuerger A., Hardes J., Goetz M. (2022). Transcriptome-wide gene expression profiles from FFPE materials based on a nuclease protection assay reveals significantly different patterns between synovial sarcomas and morphologic mimickers. Cancers.

[26] Koll F.J., Doring C., Olah C., Szarvas T., Kollermann J., Hoeh B., Chun F.K., Reis H., Wild P.J. (2023). Optimizing identification of consensus molecular subtypes in muscle-invasive bladder cancer: A comparison of two sequencing methods and gene sets using FFPE specimens. BMC Cancer.

[27] Thompson J.C., Davis C., Deshpande C., Hwang W.T., Jeffries S., Huang A., Mitchell T.C., Langer C.J., Albelda S.M. (2020). Gene signature of antigen processing and presentation machinery predicts response to checkpoint blockade in non-small cell lung cancer (NSCLC) and melanoma. J. Immunother. Cancer.

[28] Zhang W., Girard L., Zhang Y.A., Haruki T., Papari-Zareei M., Stastny V., Ghayee H.K., Pacak K., Oliver T.G., Minna J.D. (2018). Small cell lung cancer tumors and preclinical models display heterogeneity of neuroendocrine phenotypes. Transl. Lung Cancer Res..

[29] Baine M.K., Hsieh M.S., Lai W.V., Egger J.V., Jungbluth A.A., Daneshbod Y., Beras A., Spencer R., Lopardo J., Bodd F. (2020). SCLC subtypes defined by ASCL1, NEUROD1, POU2F3, and YAP1: A comprehensive immunohistochemical and histopathologic characterization. J. Thorac. Oncol..

[30] Ireland A.S., Micinski A.M., Kastner D.W., Guo B., Wait S.J., Spainhower K.B., Conley C.C., Chen O.S., Guthrie M.R., Soltero D. (2020). MYC drives temporal evolution of small cell lung cancer subtypes by reprogramming neuroendocrine fate. Cancer Cell.

[31] Owonikoko T.K., Dwivedi B., Chen Z., Zhang C., Barwick B., Ernani V., Zhang G., Gilbert-Ross M., Carlisle J., Khuri F.R. (2021). YAP1 expression in SCLC defines a distinct subtype with T-cell-inflamed phenotype. J. Thorac. Oncol..

[32] Lissa D., Takahashi N., Desai P., Manukyan I., Schultz C.W., Rajapakse V., Velez M.J., Mulford D., Roper N., Nichols S. (2022). Heterogeneity of neuroendocrine transcriptional states in metastatic small cell lung cancers and patient-derived models. Nat. Commun..

[33] Torres-Mejia E., Weng S., Nguyen K., Duong E., Yim L., Spranger S. (2023). Lung cancer-intrinsic SOX2 expression mediates resistance to checkpoint blockade therapy by inducing Treg-dependent CD8+ T cell exclusion. bioRxiv.

[34] Wu R., Wang C., Li Z., Xiao J., Li C., Wang X., Kong P., Cao J., Huang F., Li Z. (2020). SOX2 promotes resistance of melanoma with PD-L1 high expression to T-cell-mediated cytotoxicity that can be reversed by SAHA. J. Immunother. Cancer.

[35] Rudin C.M., Balli D., Lai W.V., Richards A.L., Nguyen E., Egger J.V., Choudhury N.J., Sen T., Chow A., Poirier J.T. (2023). Clinical benefit from immunotherapy in patients with SCLC is associated with tumor capacity for antigen presentation. J. Thorac. Oncol..

[36] Sabari J.K., Lok B.H., Laird J.H., Poirier J.T., Rudin C.M. (2017). Unravelling the biology of SCLC: Implications for therapy. Nat. Rev. Clin. Oncol..

[37] Steele K.E., Tan T.H., Korn R., Dacosta K., Brown C., Kuziora M., Zimmermann J., Laffin B., Widmaier M., Rognoni L. (2018). Measuring multiple parameters of CD8+ tumor-infiltrating lymphocytes in human cancers by image analysis. J. Immunother. Cancer.

[38] Ahn M.J., Cho B.C., Felip E., Korantzis I., Ohashi K., Majem M., Juan-Vidal O., Handzhiev S., Izumi H., Lee J.S. (2023). Tarlatamab for patients with previously treated small-cell lung cancer. N. Engl. J. Med..

[39] Canova S., Trevisan B., Abbate M.I., Colonese F., Sala L., Baggi A., Bianchi S.P., D’Agostino A., Cortinovis D.L. (2023). Novel therapeutic options for small cell lung cancer. Curr. Oncol. Rep..

[40] de Biasi A.R., Villena-Vargas J., Adusumilli P.S. (2014). Cisplatin-induced antitumor immunomodulation: A review of preclinical and clinical evidence. Clin. Cancer Res..

[41] Hato S.V., Khong A., de Vries I.J., Lesterhuis W.J. (2014). Molecular pathways: The immunogenic effects of platinum-based chemotherapeutics. Clin. Cancer Res..

[42] Park S.J., Ye W., Xiao R., Silvin C., Padget M., Hodge J.W., Van Waes C., Schmitt N.C. (2019). Cisplatin and oxaliplatin induce similar immunogenic changes in preclinical models of head and neck cancer. Oral Oncol..

[43] Tran L., Allen C.T., Xiao R., Moore E., Davis R., Park S.J., Spielbauer K., Van Waes C., Schmitt N.C. (2017). Cisplatin alters antitumor immunity and synergizes with PD-1/PD-L1 inhibition in head and neck squamous cell carcinoma. Cancer Immunol. Res..

[44] Yang J., Jiang J. (2024). Gasdermins: A dual role in pyroptosis and tumor immunity. Front. Immunol..

[45] Doyle A., Martin W.J., Funa K., Gazdar A., Carney D., Martin S.E., Linnoila I., Cuttitta F., Mulshine J., Bunn P. (1985). Markedly decreased expression of class I histocompatibility antigens, protein, and mRNA in human small-cell lung cancer. J. Exp. Med..

[46] Funa K., Gazdar A.F., Minna J.D., Linnoila R.I. (1986). Paucity of beta 2-microglobulin expression on small cell lung cancer, bronchial carcinoids and certain other neuroendocrine tumors. Lab. Investig..

[47] Mahadevan N.R., Knelson E.H., Wolff J.O., Vajdi A., Saigi M., Campisi M., Hong D., Thai T.C., Piel B., Han S. (2021). Intrinsic immunogenicity of small cell lung carcinoma revealed by its cellular plasticity. Cancer Discov..

[48] Yang F., Gao Y., Geng J., Qu D., Han Q., Qi J., Chen G. (2013). Elevated expression of SOX2 and FGFR1 in correlation with poor prognosis in patients with small cell lung cancer. Int. J. Clin. Exp. Pathol..

[49] Zhou B., Lin W., Long Y., Yang Y., Zhang H., Wu K., Chu Q. (2022). Notch signaling pathway: Architecture, disease, and therapeutics. Signal Transduct. Target. Ther..

[50] Nabet B.Y., Hamidi H., Lee M.C., Banchereau R., Morris S., Adler L., Gayevskiy V., Elhossiny A.M., Srivastava M.K., Patil N.S. (2024). Immune heterogeneity in small-cell lung cancer and vulnerability to immune checkpoint blockade. Cancer Cell.

[51] Hiatt J.B., Sandborg H., Garrison S.M., Arnold H.U., Liao S.Y., Norton J.P., Friesen T.J., Wu F., Sutherland K.D., Rienhoff H.Y. (2022). Inhibition of LSD1 with bomedemstat sensitizes small cell lung cancer to immune checkpoint blockade and T-cell killing. Clin. Cancer Res..

[52] Nguyen E.M., Taniguchi H., Chan J.M., Zhan Y.A., Chen X., Qiu J., de Stanchina E., Allaj V., Shah N.S., Uddin F. (2022). Targeting lysine-specific demethylase 1 rescues major histocompatibility complex class I antigen presentation and overcomes programmed death-ligand 1 blockade resistance in SCLC. J. Thorac. Oncol..

[53] Sattler M., Salgia R. (2023). LSD1-targeted therapy-a multi-purpose key to unlock immunotherapy in small cell lung cancer. Transl. Lung Cancer Res..

[54] Bald T., Landsberg J., Lopez-Ramos D., Renn M., Glodde N., Jansen P., Gaffal E., Steitz J., Tolba R., Kalinke U. (2014). Immune cell-poor melanomas benefit from PD-1 blockade after targeted type I IFN activation. Cancer Discov..

