# Supplementary material for: Predictive Signatures for Responses to Checkpoint Blockade in Small-Cell Lung Cancer in Second-Line Therapy Do Not Predict Responses in First-Line Patients
Source: Cancers (Basel). 2024 Aug 8;16(16):2795. doi: 10.3390/cancers16162795 (PMC11353197; doi:10.3390/cancers16162795)

cancers

MDPI

Table S1. Gene Signatures.

High Neuroendocrine gene set	ASCL1, BEX1, BSN, CELF3, CHGA, CHGB, RMP1, INSM1, KIF1A, KIF5C, MYT1, RTN1, RUNDC3A, SCG3, SEZ6, SH3GL2, SYN1, SYP, SYT11, SYT4, TAGLN3, TFF3, TMSB15A, TMSB15B	Zhang, 2018 [28]
Low Neuroendocrine gene set	ABCC3, AHNAK, ANXA1, CAV1, CAV2, EMP1, EPHA2, IFITM2, ITGB4, LGALS3, MYOF, PLAU, S100A10, S100A16, SLC16A5, TACSTD2, YAP1	Zhang, 2018 [28]
Notch Signaling	APH1A, ARRB1, CCND1, CUL1, DLL1, DTX1, DTX2, DTX4, FBXW11, FZD1, FZD5, FZD7, HES1, HEY1, JAG1, KAT2A, LFNG, MAML2, NOTCH1, NOTCH2, PPARD, PRKCA, PSEN2, PSENEN, SAP30, SKP1, ST3GAL6, TCF7L2, WNT2B, WNT5A	Roper, 2021 [22] Ireland, 2020 [30]
Hippo Signaling	AJUBA, YAP1, WWTR1, TEAD1, TEAD2, TEAD3	Zhang 2018 [28], Ireland 2020 [30]
Inflammation Gene Set	CCL5, CD274 (PD1), CD3d, CD8a, CXCL9, CXCL10, CXCL11, CXCL13, HAVCR2, HLA-E, IDO1, LAG3, PDCD1, PRF1, PTPRC, STAT1	Ayers 2017 [20], Thompson 2020 [24]
Hedgehog gene set	CDON, DISP2, GAS1, GLI2, GLI3, HHAT, HHIP, IFT88, KIF7, PTCH1, SMO, SSH1, SSH2	Adapted from Hallmark gene set
Hedgehog signaling	AMOT, CDK5R1,CDK6, CELSR1, CNTFR, CRMP1, DPYSL2, ETS2, GLI1, ADGRG1, HEY1, HEY2, L1CAM, LDB1, MYH9, NF1,NKX6-1, NRCAM, NRP1, NRP2, OPHN1, PLG, PML, PTCH1, RASA1,RTN1, SCG2, SLIT1, THY1, TLE1, TLE3, VEGFA,VLDLR	Hallmark gene set
T cell Gene Set	CD2, CD3D, CD3E, CD3G, CD4, CD6, CD7, CD8A, CD8B, LCK, PRF1, ZAP70	
Macrophage Gene Set	CD14, CD163, CD68, CSF1R, ITGAM, MRC1, MS4A4A	
B Cell Gene Set	BCR, BLNK, CD79A, CXCL13	
Neutrophil Gene Set	CEBPA, CSF3R, FCGR3A, FCGR3B, FUT4, ITGAX, NCF2, NCF4, S100A8, S100A9	
Antigen Presentation gene Set	B2M, CALR, CIITA, ERAP1, NLRC5, PDIA3, PSMB8, PSMB9, PSMB10, PSME1, PSME2, PSME3, TAP1, TAP2, TAPBP, TAPBPL, RFX5	Thompson, 2020 [27]
Interferon-gamma gene Set	CXCL9, CXCL10, HLA-DR, IDO1, PRF1, STAT1	
Type 1 Interferon gene Set	ACACB, BIRC3, BST2, CXCL1, DDX60, DHX58, GBP1, HERC5, IFI27, IFI44, IFI44l, IFI6, IFIH1, IFIT3, IFITM1, IRF7, ISG15, ISG20, LGALS9, MX1, OAS1, OAS2, PARP12, RASGRP3, SAMD9, SERPING1, SLC15A3, SP110, STAT1, XAF1	Bald, 2014 [54]

Table S2. Clinical Characteristics of the Patients.

Characteristics	Total (n=35)
Sex	
Male	14 (40%)
Female	21 (60%)
Age (mean)	67.1 yrs
Race	
White	20 (57%)
Black	12 (34%)
Asian	1 (3%)
Other/unknown	2 (6%)
Smoking History	
Never	1 (3%)
Current	15 (43%)
Former	19 (54%)
ECOG Performance Status	
0	4 (11%)
1	19 (54%)
2	6 (17%)
3	6 (17%)
Treatment	
First-line	21(60%)
Second-line	14 (40%)
Response	
Durable Clinical Benefit	11(34%)
No Durable Benefit	24 (66%)

Supplemental Figure S1. Characterization of Patients. The progression free survival (PFS) (in weeks) of each patient treated in second-line or first-line is plotted. The red line marks the 26 week cutoff: >26 represents durable clinical benefit (DCB). The tables show the distribution of patients. OS= overall survival.

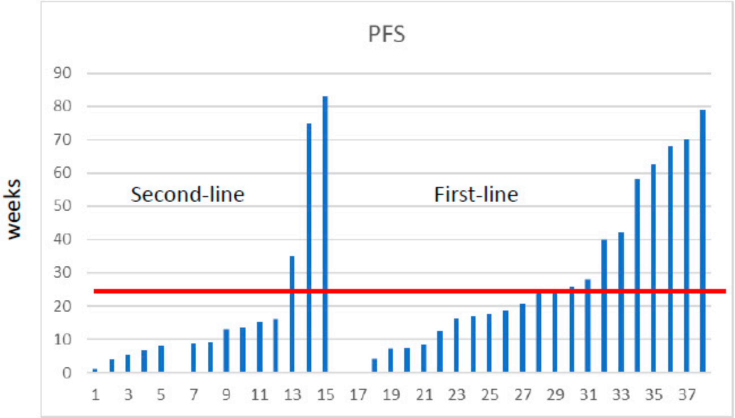

	First-Line	Second-Line	Total
NO DCB	13	11	24
DCB	8	3	11
Total	21	14	35

	First-Line	Second-Line	Total
PFS (weeks)	31	21	27
OS (weeks)	46	32	40

Supplemental Figure S2- SCLC Subtype Classification

Log2 gene expression of the 4 defining subtype genes were listed for each patient. The relative expression level for each gene is color coded (red = highest; green= lowest). Subtype was assigned according to which gene was dominant.

ASCL1	NEUROD1	YAP1	pou2F3		Subtype
10.31	5.67	3.33	-3.37		ASCL1
10.31	5.43	3.72	-5.09		ASCL1
9.37	5.36	2.22	-4.96		ASCL1
9.13	4.91	3.60	1.15		ASCL1
9.29	-2.16	4.15	1.48		ASCL1
10.01	3.68	4.01	2.37		ASCL1
11.15	4.32	3.43	-0.85		ASCL1
10.22	2.62	4.75	-5.03		ASCL1
10.38	2.05	3.75	-0.87		ASCL1
10.01	3.68	4.72	2.55		ASCL1
10.16	-0.22	4.28	-1.41		ASCL1
10.27	4.05	3.44	1.21		ASCL1
10.73	7.42	2.95	1.78		ASCL1
10.46	4.57	1.75	-4.99		ASCL1
10.15	-4.82	3.72	0.54		ASCL1
11.41	-1.14	4.80	-0.93		ASCL1
10.04	1.93	4.35	-5.10		ASCL1
10.64	1.67	3.40	-0.26		ASCL1
9.50	0.75	3.22	-1.87		ASCL1
8.89	6.00	3.86	-2.78		NeuroD1
8.03	6.61	4.59	-1.38		NeuroD1
7.65	7.13	4.30	2.18		NeuroD1
5.73	8.77	2.34	-5.02		NeuroD1
3.15	8.77	4.37	-5.00		NeuroD1
9.15	8.78	-0.75	-0.10		NeuroD1
-0.99	-2.09	3.36	6.76		Pou2F3
6.09	1.74	2.26	7.88		Pou2F3
0.53	5.53	3.91	3.64		Pou2F3
8.22	4.35	4.05	0.80		Pou2F3
7.87	-4.96	4.51	-3.37		I
9.24	-5.01	4.24	0.20		I
8.41	0.73	3.73	-4.82		I
9.66	3.98	4.62	-5.12		I
8.85	6.04	4.46	0.16		I
9.29	1.74	4.94	-5.05		I

Supplemental Figure S3. Correlations of combined neuroendocrine log2 z scores with log2 differentiation gene expression of *ASCL1, NEUROD1, YAP1*, and MYC, and the *HIPPO* log2 z score, and *NOTCH* log2 z score. Pearson correlation coefficients and p values for continuous variables are shown.

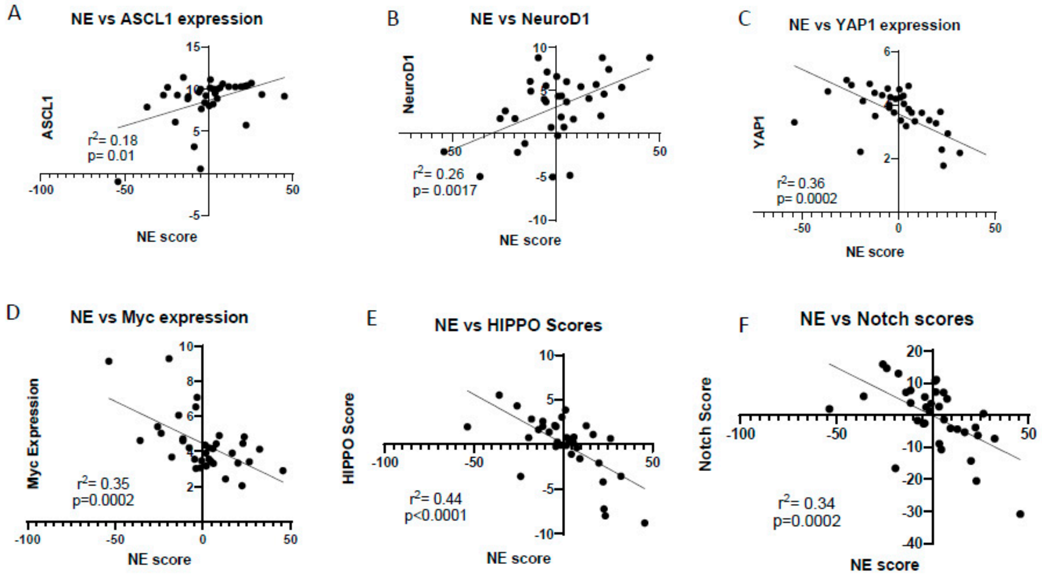

Supplemental Figure S4. Correlations of *YAP1* gene count with the (A) inflammation, (B) T cell (C), and antigen presenting machinery (APM) log2 z score. Correlation of the *HIPPO* log2 z score with the Inflammation log2 z score shown in Panel D. Pearson correlation coefficients and p values for continuous variables are shown.

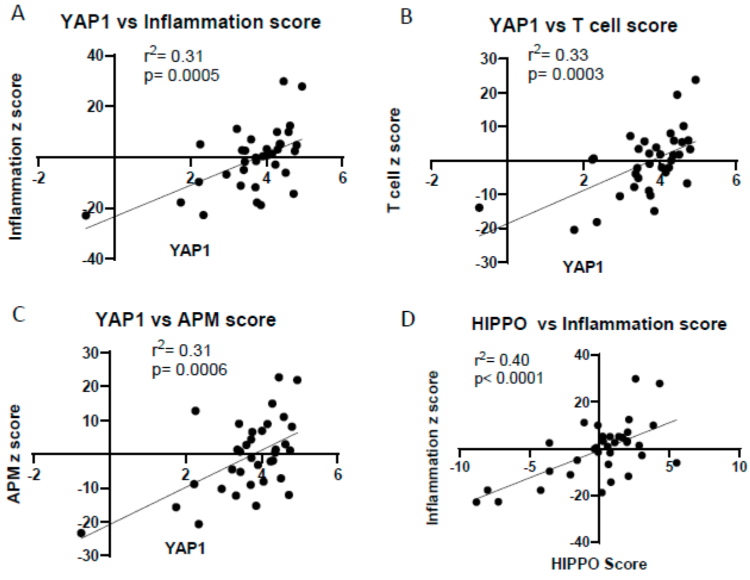

Supp. Fig S5.

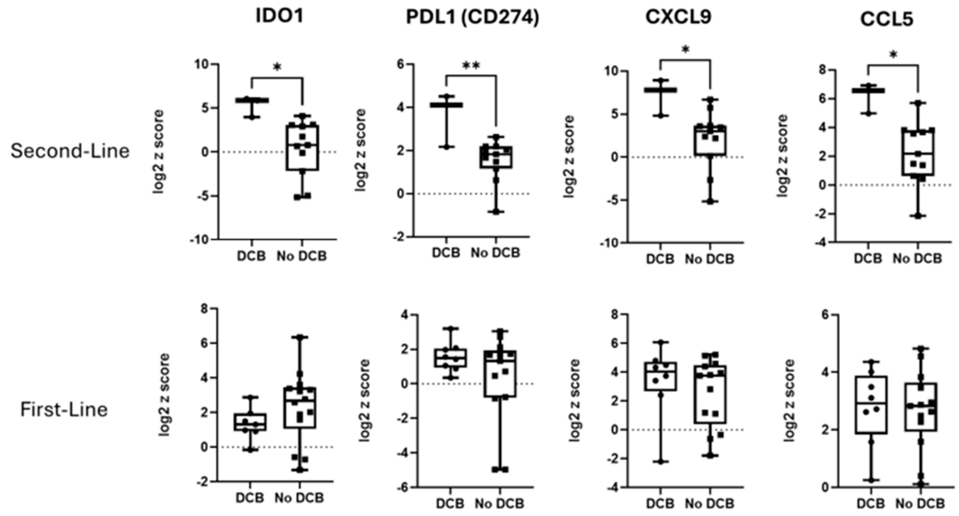

The log2 z scores of the mRNA levels of IDO1, PDL1, CXCL9, and CCL5 are plotted for patients with durable clinical benefit (DCB) or no durable clinical benefit (No DCB). The upper panels show data from from the second-line patients. The lower panels show data from the first-line patients. Values were compared using t tests. *= $p<0.05$, **=$p<0.01$.

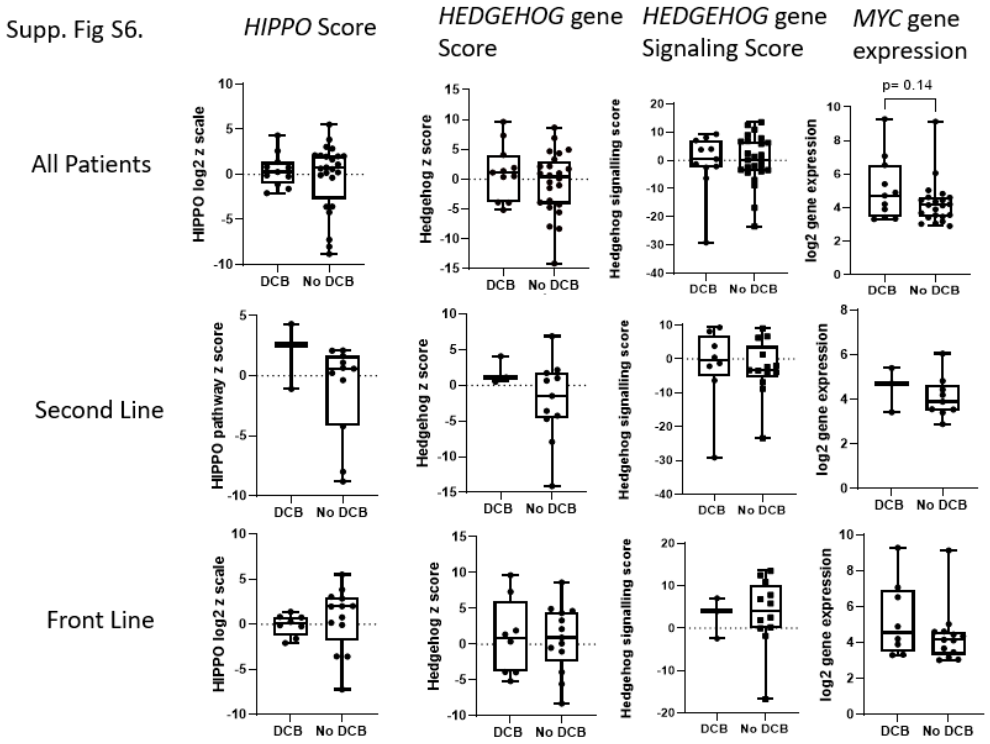

The *HIPPO* pathway log2 z score, the Hedgehog log2 gene expression score, the *HEDGEHOG* gene signaling log2 z score and the log2 of *MYC* gene expression are plotted for patients with durable clinical benefit (DCB) or no durable clinical benefit (NDB). The upper panels show data from the entire cohort of patients. The middle panel shows data from the second-line patients. The lower panels show data from the first-line patients. Values were compared using t tests. No comparisons were significant.

Supplemental Fig. S7 CD8 Staining. Biopsies were stained with an anti-CD8 antibody (red) and counterstained with hematoxylin. Examples of a highly infiltrated ("hot") tumor, a tumor with T cells in the stroma but not within the tumor ("excluded tumor"), and tumors with minimal infiltration by CD8 cells in any compartment ("cold") are shown.

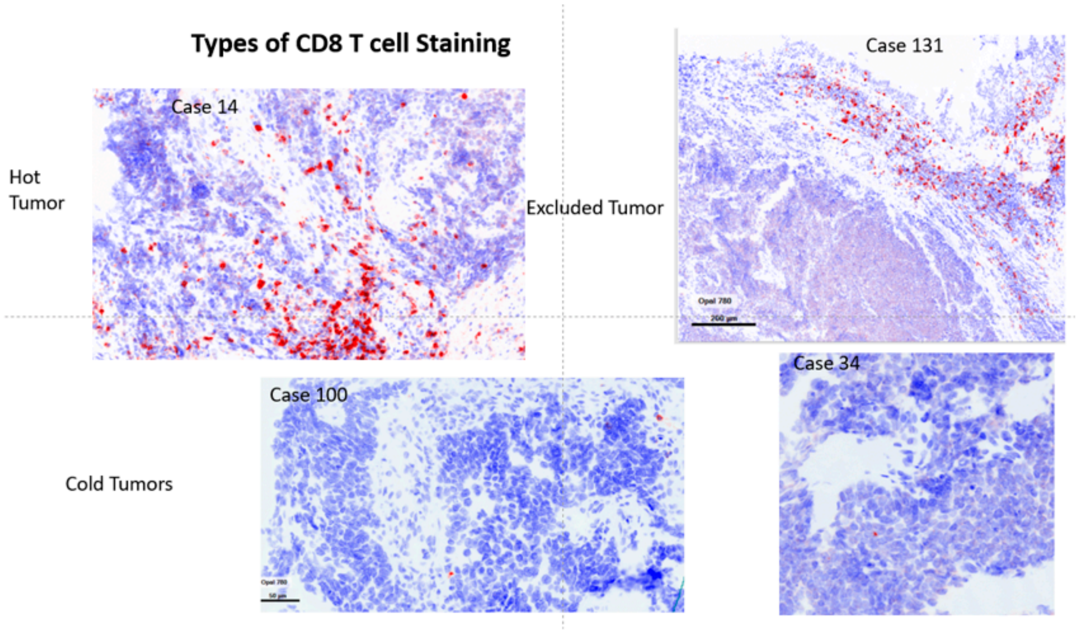

Supplement: Supplementary file 1 [file cancers-16-02795-s001.zip › cancers-3117519-supplementary.pdf]
